# Association between female infertility and stroke mortality: evidence from the PLCO cancer screening trial

**DOI:** 10.3389/fendo.2024.1433930

**Published:** 2024-09-24

**Authors:** Hui Tang, Xueming Yang, Zhou Li, Yuan Zhang, Huaxuan Chen, Mingjun Dai, Chuan Shao

**Affiliations:** ^1^ Department of Neurosurgery, Nanchong Central Hospital, The Second Clinical Medical College, North Sichuan Medical College, Nanchong, Sichuan, China; ^2^ Nanchong Institute of Cerebrovascular Diseases, Nanchong, Sichuan, China; ^3^ Sichuan Clinical Research Center for Neurological Disease, Nanchong, Sichuan, China; ^4^ Department of Neurosurgery, Chongqing General Hospital, Chongqing University, Chongqing, China

**Keywords:** infertility, stroke, mortality, female, long-term impact

## Abstract

**Objective:**

While infertility affects about 15% of women during their reproductive years, its long-term impact on stroke mortality after this period remains unclear. This study aims to investigate the association between infertility and stroke mortality in women using data from the Prostate, Lung, Colorectal, and Ovarian (PLCO) cancer screening trial.

**Methods:**

We analyzed data from 75,778 female participants aged 55–74 years with a median follow-up of 16.84 years. Cox proportional hazard models were used to estimate hazard ratios (HRs) and 95% confidence intervals (CIs) for stroke mortality, adjusting for potential confounders.

**Results:**

Among participants, 14.53% reported infertility. During follow-up, 1,159 women died from stroke. Compared to women without infertility, those with infertility had a higher risk of stroke mortality (HR 1.21, 95% CI 1.04–1.41, *p* = 0.016). This association remained statistically significant after adjusting for age, race, education level, marital status, smoking status, body mass index, history of hypertension, history of heart attack, history of diabetes mellitus, birth control pill use, hormone replacement therapy, endometriosis, first menstrual period and pregnancy history (HR 1.20, 95% CI 1.02–1.42, *p* = 0.029). Sensitivity and subgroup analyses yielded consistent results.

**Conclusion:**

The findings of this study indicate that infertility is associated with an increased risk of stroke mortality in women. Further research is needed to confirm these findings and elucidate the underlying mechanisms.

## Introduction

Stroke is a leading cause of death among women globally, with the aging population significantly contributing to the increasing incidence of this disease. In 2019, an estimated 12 million individuals suffered strokes globally, with more than 6.5 million succumbing to this devastating condition (1).

Infertility, defined as the inability to conceive after one year of unprotected, regular and timed intercourse, is increasingly recognized as a significant public health issue, affecting approximately 15% of women (2, 3). This condition is often linked to premature ovarian insufficiency, polycystic ovary syndrome (PCOS), endometriosis, and uterine fibroids, which are associated with low levels of ovarian hormones, hyperandrogenism, insulin resistance, and systemic inflammation. These factors contribute to endothelial dysfunction and promote atherosclerosis (4–6). Moreover, infertility and stroke share common risk factors, such as hypertension, obesity, and diabetes. Additionally, treatments for infertility, such as *in vitro* fertilization or hormone therapy, can increase the likelihood of blood clot formation, which may, in turn, elevate the risk of stroke (7, 8). Beyond physical health concerns, infertility can also lead to psychological distress, including depression, anxiety, and interpersonal difficulties, which may further contribute to stroke risk. The combination of these psychological factors with the underlying physiological disturbances may create a unique risk profile for stroke in infertile women. Previous research has indicated that infertility is a marker of increased long-term risk for all-cause mortality in women (9–11), as well as mortality from late-stage cancer and cardiovascular events (10–14). However, the specific relationship between infertility and stroke mortality remains inconclusive.

In this cohort study, we aim to explore the potential association between infertility and stroke mortality by utilizing data from the Prostate, Lung, Colorectal, and Ovarian (PLCO) Cancer Screening Trial. Understanding this connection is crucial for identifying high-risk individuals, developing effective preventive strategies, and uncovering the common biological mechanisms underlying both conditions.

## Methods

### Data set

The PLCO Cancer Screening Trial was a large randomized controlled trial designed to assess the impact of specific cancer screening tests on mortality rates for prostate, lung, colorectal, and ovarian cancers (15). Conducted between November 1993 and July 2001, the study recruited approximately 155,000 participants aged 42–78 years from 10 different centers across the United States, including Birmingham, Detroit, Denver, Honolulu, Marshfield, Minneapolis, Pittsburgh, Salt Lake City, St. Louis, and Washington D.C. Detailed inclusion and exclusion criteria are available on the National Cancer Institute’s website (https://cdas.cancer.gov/learn/plco/trial-summary). Participants were randomly allocated to either the intervention arm, where they underwent cancer screening tests, or the control arm, where they received standard medical care without cancer screenings. Data were collected through a baseline questionnaire detailing demographic and health-related information, including age, sex, race, education, body measurements, medical history, smoking habits, and reproductive factors such as infertility.

This study utilized data from the PLCO Cancer Screening Trial to explore the associations between infertility and stroke mortality.

### Participant screening

**Figure 1:**
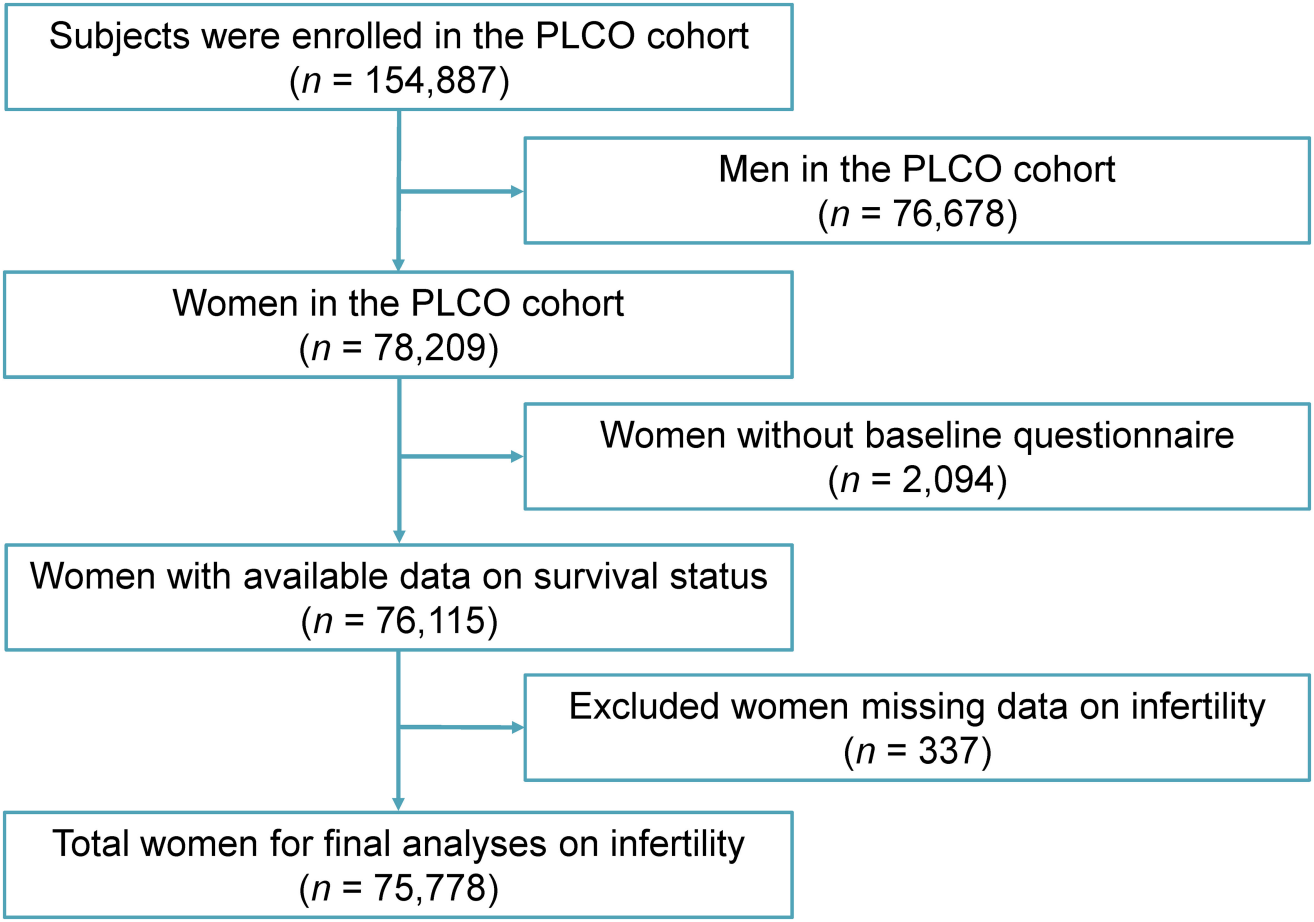
Flow chart of the screening process. PLCO, Prostate, Lung, Colorectal and Ovarian cancer screening trial.

According to the study design, 76,678 male participants were excluded, and 78,209 female participants were included. Of the female participants, 2,094 were excluded due to missing baseline questionnaires, and 337 were excluded due to missing data on infertility. After applying the inclusion and exclusion criteria, the final sample for the infertility study comprised 75,778 women, including women who later became pregnant. **Figure 1** illustrates the screening process.

### Exposure assessment

Infertility is defined as the inability to conceive after one year or more of regular, unprotected sexual intercourse. In the self-reported baseline questionnaire of the PLCO study, female infertility was assessed using the following reproductive health question: “Have you ever tried to become pregnant for a year or more without success?”. Participants who responded “yes” were classified into the “infertility” group. Participants with missing data on infertility were excluded from all analyses.

### Outcome assessment

The PLCO Cancer Screening Trial Centers implemented a multifaceted approach to ensure complete ascertainment of deaths. This included annual study update forms, reports from relatives or obituaries, and exhaustive searches of the Social Security Death Index and National Death Index for all participants. Underlying causes of death were ascertained using the 9th edition of the International Classification of Diseases from death certificates obtained from state vital statistics bureaus. To guarantee the validity of trial outcomes, a Death Review Process was established, involving a meticulous review of medical records for all decedents. This information was then used for statistical analyses of primary endpoints. Survival time was defined as the interval between randomization and either stroke-related death or the pre-defined follow-up cutoff date.

### Statistical analysis

All statistical evaluations were performed utilizing R software (version 4.1.3). The baseline attributes were expressed as counts and percentages for categorical variables, which were compared using the chi-square test. Continuous variables were represented as median (interquartile range, IQR) and compared using the Kruskal-Wallis test.

Women without reported infertility were designated as the reference group, while those reporting infertility were considered as the exposed group. Stroke death was designated as the primary outcome. We first performed univariable survival analysis to assess the association between infertility and stroke mortality, using the Kaplan-Meier method to estimate the survival probability curves. Multivariable analysis was based on a Cox proportional hazards regression model, adjusted for age, race, marital status, education level, smoking status, body mass index, history of hypertension, history of heart attack, history of diabetes mellitus, birth control pill use, hormone replacement therapy, endometriosis, first menstrual period and pregnancy history. We constructed multiple multivariable models to perform sensitivity analyses. Additionally, this study carries out subgroup analyses, accounting for potential confounding factors, to explore the relationship between infertility and stroke mortality, with the goal of assessing result consistency.

Covariates were selected based on prior literature examining associations with infertility and cardiovascular health (14, 16, 17). Age (continuous, in years) was adjusted alongside the following baseline variables: race (white or other), education level (below or at least university degree), marital status (ever married/living with partner or never married), smoking status (never, former or current), body mass index (BMI) categories (underweight/normal [less than 25kg/m²], overweight [25 to less than 30kg/m²], obese [greater than or equal to 30kg/m²]), history of hypertension (yes or no), history of heart attack (yes or no), history of diabetes mellitus (yes or no), birth control pill use (yes or no), hormone replacement therapy (yes or no), endometriosis (yes or no), first menstrual period (≤11 years, 12–13 years, or ≥14 years) and pregnancy history (yes or no). Subgroup analyses were conducted based on these potential confounders to evaluate the consistency of results. All tests were two-sided with a significance level of α = 0.05.

## Results

Among 75,778 participants, 14.53% (*n* = 11,004) self-reported infertility, while 85.47% (*n* = 64,774) reported no infertility. The baseline characteristics of the participants are summarized in **Table 1**. During a follow-up period of 16.84 (14.77–18.78) years, 17,209 women died, including 1,159 from stroke and 16,050 from other causes (**Supplementary Table 1**).

**Table 1:** Baseline characteristics of the subjects by the history of infertility at baseline.

Characteristic	Unexposed *n*, % or Mean (IQR)	Infertility *n*, % or Mean (IQR)	*p* value
Sample size	64,774 (85.47)	11,004 (14.53)	
Age (year)	62 (58, 67)	62 (57, 67)	0.368
Race			<0.001
White	57,190 (88.29)	9,978 (90.68)	
Other^#^	7,584 (11.71)	1,026 (9.32)	
Marital status			<0.001
Ever married or living with partner	62,138 (96.07)	10,974 (99.85)	
Never married	2,543 (3.93)	16 (0.15)	
Missing	93	14	
Education level			<0.001
Under university	30,809 (47.66)	4,706 (42.84)	
At least university	33,836 (52.34)	6,280 (57.16)	
Missing	129	18	
Smoking status			<0.001
Never	36,215 (55.91)	5,956 (54.13)	
Current	6,296 (9.72)	1,054 (9.58)	
Former	22,258 (34.37)	3,994 (36.30)	
Missing	5	0	
Body mass index (kg/m^2^)			0.001
Underweight or normal	26,219 (41.04)	4,627 (42.54)	
Overweight	21,954 (34.36)	3,734 (34.33)	
Obesity	15,715 (24.60)	2,516 (23.13)	
Missing	886	127	
Hypertension			0.525
No	42,516 (65.98)	7,258 (66.29)	
Yes	21,924 (34.02)	3,691 (33.71)	
Missing	334	55	
Heart attack			0.186
No	61,303 (95.20)	10,381 (94.91)	
Yes	3,090 (4.80)	557 (5.09)	
Missing	381	66	
Diabetes mellitus			0.063
No	60,343 (93.66)	10,201 (93.19)	
Yes	4,082 (6.34)	745 (6.81)	
Missing	349	58	
Birth control pill use			<0.001
No	29,239 (45.17)	5,399 (49.11)	
Yes	35,493 (54.83)	5,594 (50.89)	
Missing	42	11	
Hormone replacement therapy			<0.001
No	22,050 (34.21)	3,282 (29.97)	
Yes	42,408 (65.79)	7,668 (70.03)	
Missing	316	54	
Endometriosis			<0.001
No	57,220 (92.76)	8,878 (84.71)	
Yes	4,464 (7.24)	1,602 (15.29)	
Missing	3,090	524	
First menstrual period (year)			0.003
≤11	13,026 (20.14)	2,381 (21.67)	
12-13	34,862 (53.91)	5,797 (52.75)	
≥14	16,779 (25.95)	2,812 (25.58)	
Missing	107	14	
Pregnancy history			<0.001
No	4,036 (6.23)	1,629 (14.88)	
Yes	60,698 (93.77)	9,321 (85.12)	
Missing	40	54	

Continuous variables are presented as median (IQR, interquartile range), while categorical variables are presented as counts or percentages. ^#^Other includes Black, Hispanic, Asian, Pacific Islander, American Indian.

**Figure 2:**
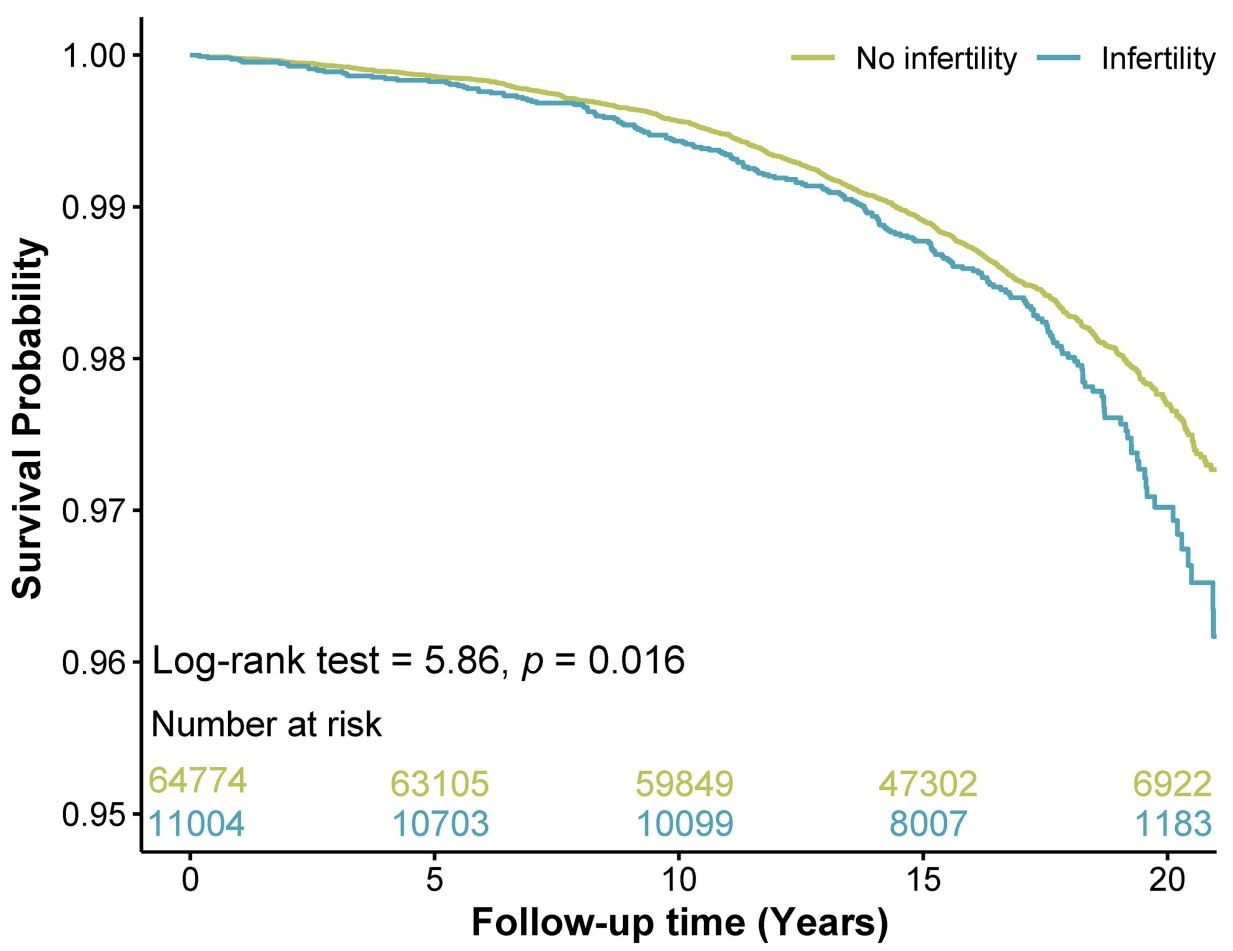
Kaplan-meier survival curves for stroke mortality in women with and without infertility. The cyan line represented the survival rate of women with infertility, and the yellow line represented the survival rate of women without infertility. The survival table details the number of women available for analysis at each time point.

**Figure 3:**
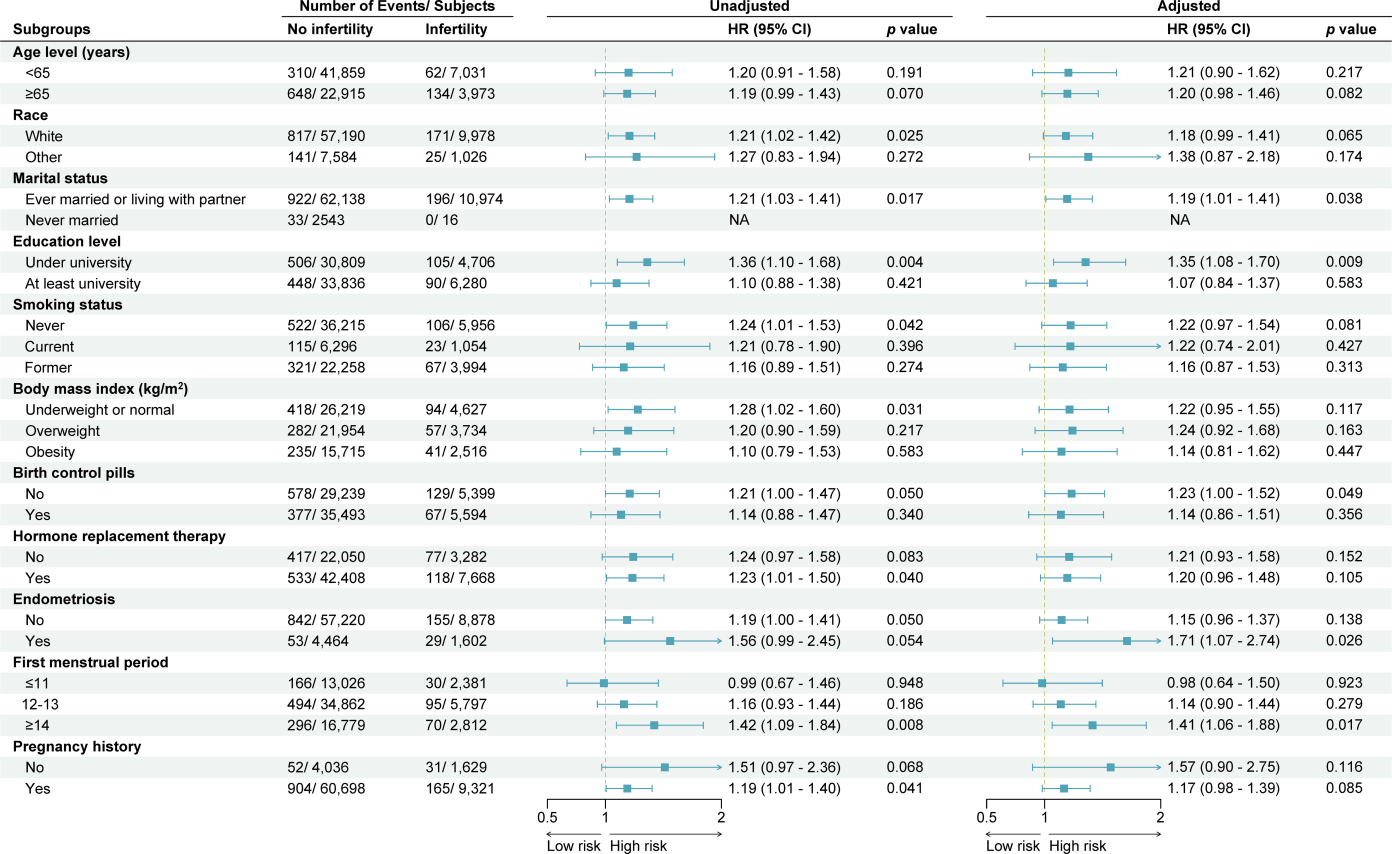
Subgroup analysis and forest plot for stroke-cause mortality in women with and without infertility. This forest plot shows the number of deaths and subjects, unadjusted and adjusted hazard ratios (HR) with 95% confidence interval (CI), according to baseline characteristics. Hazard ratios were adjusted for age, race, marital status, education level, smoking status, body mass index, history of hypertension, history of heart attack, history of diabetes mellitus, birth control pill use, hormone replacement therapy, endometriosis, first menstrual period and pregnancy history. NA, not available.

Kaplan-Meier survival analysis was utilized to assess the survival disparity between infertile and non-infertile women. The findings revealed a statistically significant difference in survival probability between the two groups, as illustrated in **Figure 2**. **Table 2** presents the results of unadjusted and adjusted analyses for the hazard ratios of stroke mortality. Unadjusted analysis showed that women with infertility had a higher risk of stroke death than those without infertility (HR 1.21, 95% CI 1.04–1.41, *p* = 0.016). After adjusting for potential confounders, the associations were slightly attenuated but remained statistically significant (HR 1.20, 95% CI 1.02–1.42, *p* = 0.029). Sensitivity analysis was conducted using multiple models incorporating different confounding factors, and the findings remained consistent (**Table 2**). Subgroup analyses based on pre-defined confounding factors suggested no significant differences in the results (**Figure 3**).

**Table 2:** Unadjusted and adjusted hazard ratios of the association between infertility and stroke mortality.

Infertility	Events (*n*)	Subjects (*n*)	Unadjusted HR (95% CI)	*p* value	Model 1 HR (95% CI)	*p* value	Model 2 HR (95% CI)	*p* value	Model 3 HR (95% CI)	*p* value	Model 4 HR (95% CI)	*p* value
**No**	958	64,774	Reference		Reference		Reference		Reference		Reference	
**Yes**	196	11,004	1.21 (1.04–1.41)	0.016	1.19 (1.02–1.38)	0.030	1.19 (1.02–1.39)	0.028	1.19 (1.01–1.39)	0.033	1.20 (1.02–1.42)	0.029

Model 1 was adjusted for age.

Model 2 was additionally adjusted for race, education level and marital status.

Model 3 was further adjusted for smoking status, body mass index, history of hypertension, history of heart attack and history of diabetes mellitus.

Model 4 was further adjusted for birth control pill use, hormone replacement therapy, endometriosis, first menstrual period and pregnancy history.

A *p* value of less than 0.05 was the criterion for statistical significance. HR, hazard ratio; CI, confidence interval.

## Discussion

In the PLCO Cancer Screening Trial study of 78,209 women with a median follow-up of 16.84 years, 1,159 died of stroke. Our study found that women with infertility had a higher risk of stroke death compared to women without infertility.

Prior studies investigating the association between infertility and stroke incidence have yielded mixed results, with some studies reporting an increased risk of stroke in infertile women (10, 18), while others have found no such association (14, 19, 20). Two studies examined mortality in relation to infertility, with consistent findings indicating an elevated all-cause mortality risk in infertile women, while demonstrating no association with cardiovascular disease mortality (10, 11). Notably, no studies have exclusively investigated the relationship between infertility and stroke mortality. Our present analysis using PLCO Cancer Screening Trial data reveals a 20% higher risk of stroke mortality in infertile women.

Our findings contrast with other studies that found no significantly increased risk of mortality from cardiovascular diseases in infertile women. Several possible explanations may account for for this discrepancy. First, the two previous studies failed to differentiate between stroke, ischemic heart disease, and other circulatory system diseases. Remarkably, approximately three-quarters of all strokes occur in individuals aged 65 years or older. Short follow-up periods may result in an underestimation of stroke mortality. Therefore, further research is needed to clarify this issue.

Several mechanisms might explain our findings. Although transient hormonal fluctuations are common among women, infertile women often experience chronic and persistent hormonal imbalances. These sustained imbalances lead to prolonged vascular dysfunction, which significantly elevated risk of stroke (5). Furthermore, specific causes of female infertility, such as endometriosis and PCOS (21), which were also found to be associated with stroke risk (4, 22–26). Endometriosis induces systemic inflammation through pro-inflammatory cytokines such as IL-1β, IL-6, and TNF-α (23). This chronic inflammatory state can induce a hypercoagulable environment, thereby increasing the risk of cardiovascular complications, such as stroke (5, 27). Our data indicate a significantly higher prevalence of endometriosis among women with infertility (15.29%) compared to those without (7.24%). Further subgroup analysis by history of endometriosis showed women with endometriosis have a significantly higher risk of stroke mortality compared to the general population. PCOS can induce insulin resistance, promoting atherosclerosis and platelet aggregation, all of which elevate ischemic stroke risk (5, 28). Infertility treatments, such as *in vitro* fertilization or hormone therapy, can induce a hypercoagulable state, increasing the risk of thrombosis and subsequently stroke (7, 8). Due to the absence of data on PCOS and infertility treatments in PLCO study, the impact of PCOS-related infertility and infertility treatments on stroke mortality remains unconfirmed in the current analysis. Therefore, further research should focus more specifically on identifying the underlying causes of female infertility and their potential link to stroke mortality.

This study leverages a large prospective cohort with long-term follow-up, strengthening its generalizability. However, some limitations also exist in the current study. First, the reliance on self-reported data from a retrospective questionnaire on infertility introduces potential bias. Second, the lack of data on male partner fertility may lead to an overestimation of the association between female infertility and stroke mortality, as infertility can be attributed to factors from either or both partners. Third, the absence of detailed stroke subtype and fertility treatment data in the PLCO study impedes the differentiation between hemorrhagic and ischemic stroke mortality and hampers the assessment of risks associated with fertility treatment status. Lastly, this study focused on individuals within the U.S., indicating that future research in other geographic regions is necessary to validate and generalize these findings.

## Conclusions

Our findings suggest that infertility might be a potential risk factor for stroke mortality in women, potentially serving as an early indicator of long-term cerebrovascular health. Well-designed prospective cohort studies are crucial to validate these observations. Further research is warranted to elucidate the underlying mechanisms linking infertility and stroke mortality.

## Data Availability

The data analyzed in this study is subject to the following licenses/restrictions: While the current study utilized data from the National Cancer Institute PLCO study group under a data use license, accessing this dataset requires following specific procedures. Requests to access these datasets should be directed to https://cdas.cancer.gov/plco.
