# Corrigendum: Association between female infertility and stroke mortality: evidence from the PLCO cancer screening trial

**DOI:** 10.3389/fendo.2024.1510810

**Published:** 2024-11-05

**Authors:** Hui Tang, Xueming Yang, Zhou Li, Yuan Zhang, Huaxuan Chen, Mingjun Dai, Chuan Shao

**Affiliations:** ^1^ Department of Neurosurgery, Nanchong Central Hospital, The Second Clinical Medical College, North Sichuan Medical College, Nanchong, Sichuan, China; ^2^ Nanchong Institute of Cerebrovascular Diseases, Nanchong, Sichuan, China; ^3^ Sichuan Clinical Research Center for Neurological Disease, Nanchong, Sichuan, China; ^4^ Department of Neurosurgery, Chongqing General Hospital, Chongqing University, Chongqing, China

**Keywords:** infertility, stroke, mortality, female, long-term impact

In the published article, there was an error in **Table 1** and **Figure 3** as published. The terms ‘Former’ and ‘Current’ were inadvertently transposed within the smoking status category. The corrected **Table 1** and **Figure 3** and its caption appear below.

**Table 1:** Baseline characteristics of the subjects by the history of infertility at baseline.

Characteristic	Unexposed*n*, % or Mean (IQR)	Infertility*n*, % or Mean (IQR)	*p* value
Sample size	64,774 (85.47)	11,004 (14.53)	
Age (year)	62 (58, 67)	62 (57, 67)	0.368
Race			<0.001
White	57,190 (88.29)	9,978 (90.68)	
Other ^#^	7,584 (11.71)	1,026 (9.32)	
Marital status			<0.001
Ever married or living with partner	62,138 (96.07)	10,974 (99.85)	
Never married	2,543 (3.93)	16 (0.15)	
Missing	93	14	
Education level			<0.001
Under university	30,809 (47.66)	4,706 (42.84)	
At least university	33,836 (52.34)	6,280 (57.16)	
Missing	129	18	
Smoking status			<0.001
Never	36,215 (55.91)	5,956 (54.13)	
Current	6,296 (9.72)	1,054 (9.58)	
Former	22,258 (34.37)	3,994 (36.30)	
Missing	5	0	
Body mass index (kg/m^2^)			0.001
Underweight or normal	26,219 (41.04)	4,627 (42.54)	
Overweight	21,954 (34.36)	3,734 (34.33)	
Obesity	15,715 (24.60)	2,516 (23.13)	
Missing	886	127	
Hypertension			0.525
No	42,516 (65.98)	7,258 (66.29)	
Yes	21,924 (34.02)	3,691 (33.71)	
Missing	334	55	
Heart attack			0.186
No	61,303 (95.20)	10,381 (94.91)	
Yes	3,090 (4.80)	557 (5.09)	
Missing	381	66	
Diabetes mellitus			0.063
No	60,343 (93.66)	10,201 (93.19)	
Yes	4,082 (6.34)	745 (6.81)	
Missing	349	58	
Birth control pill use			<0.001
No	29,239 (45.17)	5,399 (49.11)	
Yes	35,493 (54.83)	5,594 (50.89)	
Missing	42	11	
Hormone replacement therapy			<0.001
No	22,050 (34.21)	3,282 (29.97)	
Yes	42,408 (65.79)	7,668 (70.03)	
Missing	316	54	
Endometriosis			<0.001
No	57,220 (92.76)	8,878 (84.71)	
Yes	4,464 (7.24)	1,602 (15.29)	
Missing	3,090	524	
First menstrual period (year)			0.003
≤11	13,026 (20.14)	2,381 (21.67)	
12-13	34,862 (53.91)	5,797 (52.75)	
≥14	16,779 (25.95)	2,812 (25.58)	
Missing	107	14	
Pregnancy history			<0.001
No	4,036 (6.23)	1,629 (14.88)	
Yes	60,698 (93.77)	9,321 (85.12)	
Missing	40	54	

Continuous variables are presented as median (IQR, interquartile range), while categorical variables are presented as counts or percentages. ^#^Other includes Black, Hispanic, Asian, Pacific Islander, American Indian.

**Figure 3:**
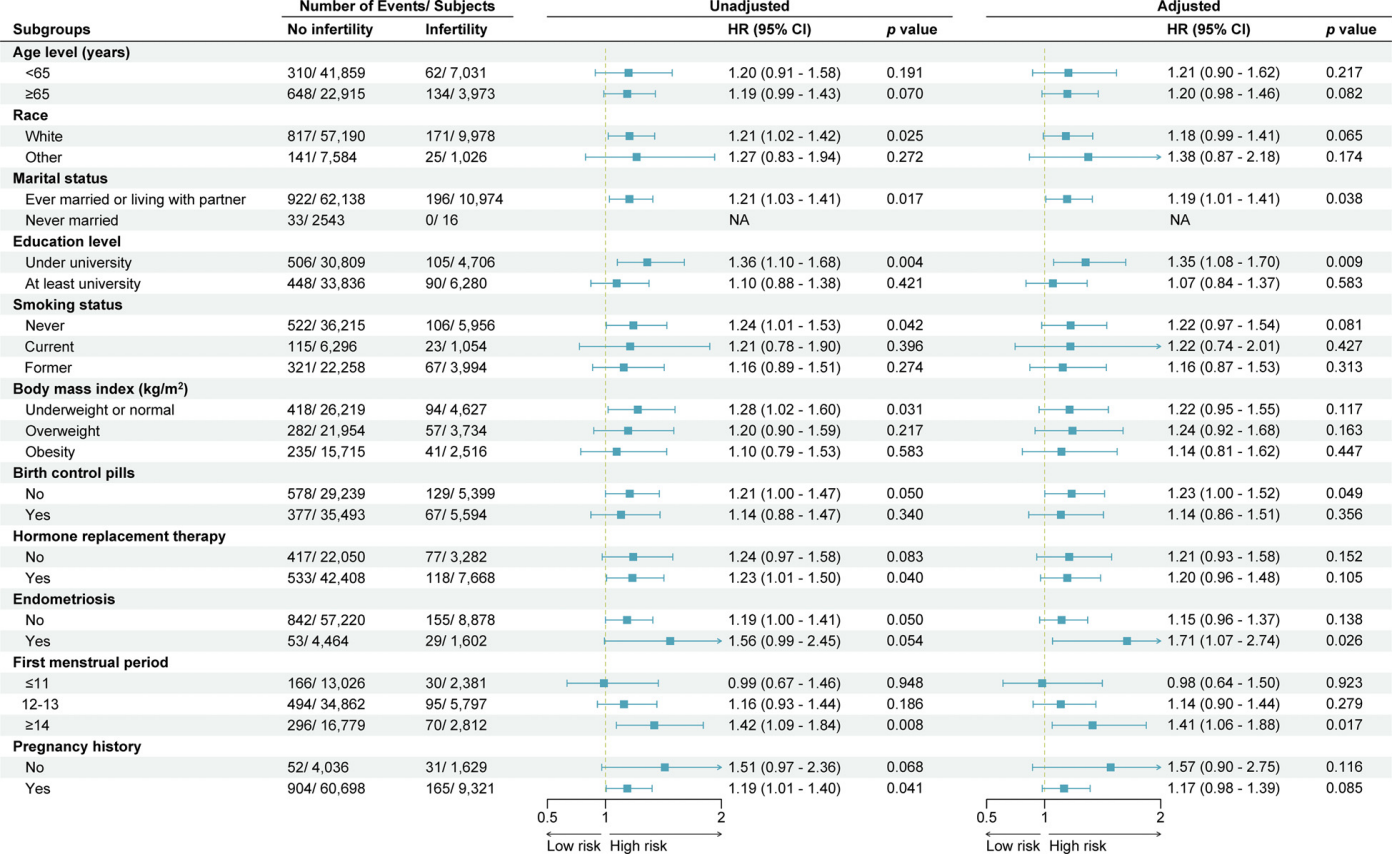
Subgroup analysis and forest plot for stroke-cause mortality in women with and without infertility. This forest plot shows the number of deaths and subjects, unadjusted and adjusted hazard ratios (HR) with 95% confidence interval (CI), according to baseline characteristics. Hazard ratios were adjusted for age, race, marital status, education level, smoking status, body mass index, history of hypertension, history of heart attack, history of diabetes mellitus, birth control pill use, hormone replacement therapy, endometriosis, first menstrual period and pregnancy history. NA, not available.

The authors apologize for this error and state that this does not change the scientific conclusions of the article in any way. The original article has been updated.

