# Supplementary material for: Association between female infertility and stroke mortality: evidence from the PLCO cancer screening trial
Source: Front Endocrinol (Lausanne). 2024 Sep 24;15:1433930. doi: 10.3389/fendo.2024.1433930 (PMC11458404; doi:10.3389/fendo.2024.1433930)
Supplement: Supplementary file 1 [file Table1.pdf]

Supplementary Material

Supplementary Table 1. A summary table of all-causes and specific causes of death.

**Causes of death in women**	**Number of deaths (%)**
Total deaths	17,209 (100)
Stroke	1,159 (6.74)
Accident	573 (3.33)
Cancer	5,540 (32.19)
Digestive disease	507 (2.95)
Other diseases of the nervous system	1,028 (5.97)
Endocrine, nutritional, metabolic diseases and immunity disorders	583 (3.39)
Infectious disease	363 (2.11)
Ischemic heart disease	1,673 (9.72)
Other circulatory diseases	2,272 (13.20)
Respiratory illness	1,656 (9.62)
Other	1,855 (10.78)

The causes of death among women were categorized into eleven distinct groups based on the International Classification of Diseases, Ninth Edition. Data are presented as numbers (%).
